# Mapping the global research landscape on depression for patients with chronic kidney disease: a bibliometric and visualized analysis from 2006 to 2022

**DOI:** 10.3389/fphar.2024.1388641

**Published:** 2024-07-17

**Authors:** Wenchao Xu, Zitong Chen, Yurong Zhang, Jiayu Zhao, Wenyong Huang, Xiao Guo, Jianyu Zhang

**Affiliations:** ^1^ Department of Rheumatology and Immunology, The Third Affiliated Hospital, Guangzhou Medical University, Guangzhou, China; ^2^ Guangdong Provincial Key Laboratory of Major Obstetric Diseases, Guangzhou, China; ^3^ Guangdong Provincial Clinical Research Center for Obstetrics and Gynecology, Guangzhou, China; ^4^ State Key Laboratory of Ophthalmology, Zhongshan Ophthalmic Center, Sun Yat-sen University, Guangzhou, China; ^5^ Guangdong Provincial Key Laboratory of Ophthalmology and Visual Science, Guangzhou, China; ^6^ Guangdong Provincial Clinical Research Center for Ocular Diseases, Guangzhou, China; ^7^ The Third Affiliated Hospital of Jinzhou Medical University, Jinzhou, China

**Keywords:** depression, bibliometrix, VOSviewer, CKD, chronic kidney disease

## Abstract

**Background:**

Chronic Kidney Disease (CKD), a complex and multifaceted health issue, significantly contributes to global mortality rates. Accompanying chronic conditions, depression notably exacerbates health outcomes, increasing both mortality risk and the burden on affected individuals. This study employs bibliometric and visual analytics to evaluate the evolution, current trends, and future research directions in the field of CKD and depression.

**Methods:**

We conducted a thorough investigation using the Web of Science Core Collection, focusing on literature published from 2006 to 2022 that examines the interplay between CKD and depression. The analysis was enriched with bibliometric and visualization tools such as bibliometrix, CiteSpace, and VOSviewer to distill the essence of the research corpus.

**Results:**

Our analysis incorporated 2,409 CKD-related publications, with significant contributions from the United States, China, and England. BMC Nephrology emerged as the leading publication outlet, while the American Journal of Kidney Diseases featured the most cited articles. Key terms such as “depression,” “quality-of-life,” “mortality,” “prevalence,” and “hemodialysis” dominated the keyword landscape, indicating the research focus areas.

**Conclusion:**

This bibliometric analysis offers an in-depth view of the research trajectory in CKD and depression. It provides valuable insights for researchers seeking relevant literature, potential collaborators, and an understanding of the field’s current hotspots and emerging frontiers. The findings of this study are instrumental in guiding and enriching future research endeavors in this domain.

## Introduction

Chronic Kidney Disease (CKD) is a global health challenge that affects >10% of the general population worldwide, amounting to >800 million individuals (Kovesdy, 2011; Bikbov et al., 2020). Characterized by the gradual loss of kidney function, CKD encompasses a spectrum of diseases that collectively contribute significantly to global morbidity and mortality (Lv and Zhang, 2019). Alongside physical ailments, CKD is often accompanied by psychological complications, dementia and cognitive dysfunction, among which depression is notably prevalent (Kogon and Hooper, 2023). A number of recent large and well-conducted studies have confirmed significantly increased rates of depression in patients with CKD, with a meta-analysis suggesting a prevalence of interview-defined depression of approximately 20% (Bautovich et al., 2014). Depression, a serious mental health condition, can profoundly impact the quality of life and overall wellbeing of individuals, particularly those grappling with chronic illnesses like CKD. The intersection of CKD and depression presents a complex clinical picture, demanding a nuanced understanding of their interrelation for effective management and treatment (Liu M. et al., 2022).

Understanding the relationship between CKD and depression is critical for healthcare providers, researchers, and policymakers aiming to improve the quality of life for people with CKD and depression. The relationship between CKD and depression is intricate and bidirectional (Liu M. et al., 2022). On the one hand, the physical, emotional, and social burdens of living with a chronic condition like CKD can precipitate or exacerbate depressive symptoms (Taylor et al., 2016; Jia et al., 2020). On the other hand, depression can adversely impact the course of CKD, potentially influencing disease progression and treatment adherence (Bautovich et al., 2014). Despite the recognition of this interplay, gaps remain in the comprehensive understanding of how these conditions influence one another. A thorough exploration of existing literature is essential to bridge these gaps, offering insights into effective management strategies that address both the physical and psychological aspects of CKD.

Bibliometric analysis is a quantitative method of publication research. It involves conducting co-occurrence analysis, social network analysis, cluster analysis and other techniques to summarize the progress of a research topic and identify emerging trends and contributions from authors, journals, institutions or countries (Liu et al., 2021; Liu Y. et al., 2022). With the advent of scientific databases such as Web of Science, research data are now easily accessible, which has facilitated the development of bibliometric studies. Several researchers have examined the current research and clinical applications of the relationship between CKD and chronic malnutrition, uremic cardiomyopathy and SARS-CoV-2 infection utilizing bibliometric analysis (Wang et al., 2023). However, there is no bibliometric analyses of CKD and depression have been published together. To fill this gap, this bibliometric analysis constructs a global map of scientific publications on CKD and depression related research.

## Materials and methods

### Data collection and strategy for data retrieval

**FIGURE 1:**
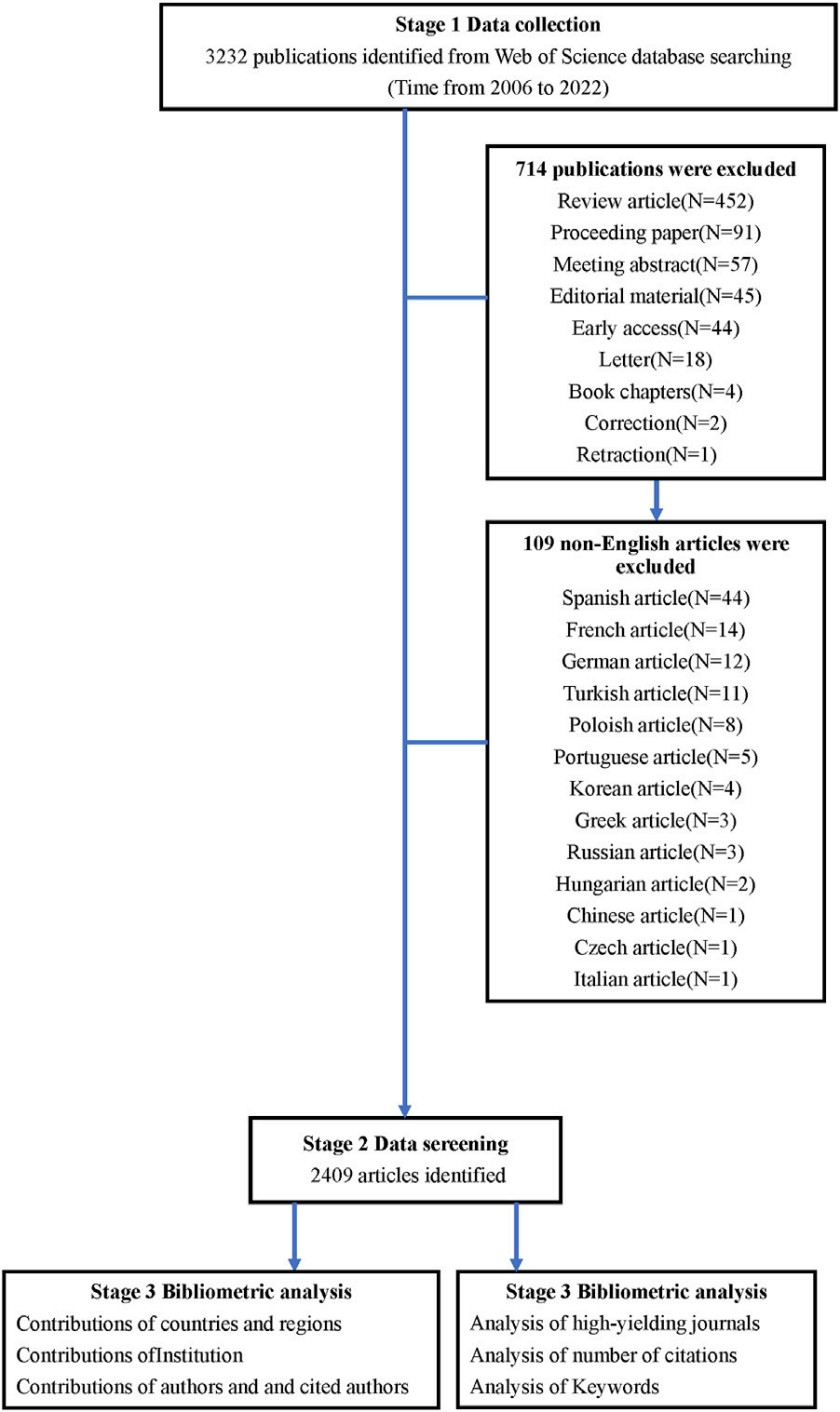
The data collection and retrieval strategy.

We conducted a comprehensive search on web of science, retrieving publications from 2006 to 2022. We used the search phrases “TS = (chronic kidney disease) OR TS = (CKD) OR TS = (chronic renal disease) OR TS = (chronic renal failure) OR TS = (chronic renal insufficiency) OR TS = (lupus nephritis) OR TS = (chronic glomerulonephritis) OR TS = (chronic nephropathy) OR TS = (chronic nephritis) OR TS = (tubulointerstitial nephritis) OR TS = (diabetic kidney) OR TS = (diabetic nephropathy) OR TS = (nephrotic syndrome) AND TS = (depression) OR TS = (depressive symptoms) OR TS = (emotional depression)” to retrieve all relevant records. The search timespan covered the years 2006–2022, a period we considered long enough to reflect the development trends in the field we focused on. All relevant records were extracted and imported to Bibliometrix and VOSviewer for bibliometric analysis. The retrieved publications had to satisfy the following criteria: 1) the document type was “article”; 2) the article was in English; 3) the following information was collected: publication, authors, countries, institutions, journals, keywords, and citations. The retrieved data were collected within 1 day to avoid any potential deviations due to daily updates. A total of 3,372 publications were retrieved, of which 963 irrelevant publications were excluded, including review articles, proceeding papers, meeting abstracts, editorial materials, early accesses, letters, book chapters, corrections, retractions and non-English articles. Figure 1 shows the flowchart for data selection.

### Data analysis and network mapping

Bibliometric analysis was conducted utilizing R version 3.5.6, VOSviewer, and CiteSpace. Bibliometrix is an R package that contains a range of features for quantitative scientometrics research. In this study, it allowed for the determination of the highest cumulative occurrences of keywords/terms per year, calculation of the frequency of collaborations between countries, and visualization of keywords while analyzing the three domains of the triple-base map. Co-authorship analysis reveals patterns of collaboration between authors, institutions and countries. Co-occurrence analysis uses the frequency of occurrence of multiple words in the same article to identify their proximity to each other, demonstrating hot topics and trends in the discipline. Co-citation analysis helps researchers discover and identify the knowledge base of the discipline.

CiteSpace is a Java application that generates dynamic visualizations to reflect the evolution of bibliometric networks over time. The study identified the most significant citation bursts for highly cited references and keywords over time.

Furthermore, a generalized additivity model implemented through the R of the mgcv package was used to estimate the trend and count of publications. To visualize international cooperation between countries, an online bibliometrics website (https://bibliometric.com/) was employed.

In this study, we used VOSviewer to integrate all the collected literature, from which we extracted information about literature titles, information on authors, research institutions, countries/regions, citations and keywords to create a visual network map. In the VOS-Viewer visualization map, each node is represented by a labelled circle. Larger circles appear more often in co-occurrence analysis. The color of each circle is determined by the cluster to which it belongs. The thickness and length of the links between nodes represent the strength and relevance of the connections between the corresponding nodes.

## Results

### Publication overview

**FIGURE 2:**
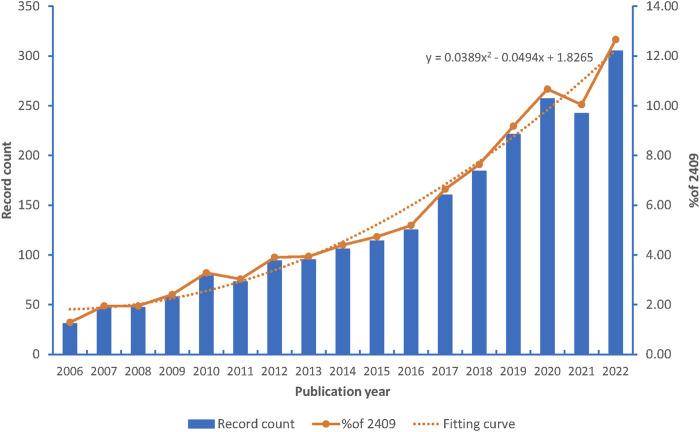
Number of publications by year and curve fitting of the annual growth trend of publications.

A comprehensive search of the Web of Science database for literature on CKD published between 2006 and 2022 revealed a total of 2,409 articles (Figure 1). The annual publication trends are visually depicted in Figure 2, showcasing a remarkable surge in interest over the past 16 years. The global annual number of publications surged from 47 in 2006 to an impressive 305 in 2022, marking an exceptional increase of 548.9%. Noteworthy shifts in publication dynamics are observed when examining specific timeframes. From 2006 to 2013, the annual publications remained below 100. However, a substantial and consistent upward trajectory is evident from 2014 to 2020, with the annual publications rising from 106 to 257. Particularly noteworthy is the surge in articles, exceeding 300 in 2022, reaching a peak at 305. Figure 2 illustrates the fitting curve for the overall annual growth trend of publications, further emphasizing the escalating attention directed toward CKD research. These findings underscore the escalating interest and attention dedicated to the research on CKD over the years.

### Distribution and co-authorship of authors

A total of 12,761 authors, making 913 appearances, collectively contributed to the 2,409 retrieved articles, with an average of 5.3 authors per article. Notably, Table 1 showcases the top 10 most prolific authors in CKD and depression publications. Paul L. Kimmel stands out as the most productive, with an impressive record of 23 publications and 1,926 citations. Following closely are Steven D. Weisbord (19 publications, 1,045 citations) and Marta Novak (19 publications, 649 citations). Examining the references of an article provides insights into author contributions, and Table 2 highlights the top 10 most frequently cited authors. These influential figures have amassed over 600 citations each, with Paul L. Kimmel leading the pack with 1,926 citations, followed by RA Peterson (1,221) and Kimmel PL (1,179). Paul L. Kimmel, a distinguished professor of Clinical Medicine at George Washington University, emerges as a trailblazer in CKD research. His remarkable contributions span the biological basis, clinical pathophysiology, and treatment of CKD. Professor Kimmel’s diverse research interests encompass sleep disorders in kidney disease patients, zinc metabolism in kidney disease, AIDS nephropathy, psychological and social adaptation to CKD, clinical genetics of prevalent kidney diseases, and the correlation between acute kidney injury and CKD.

**TABLE 1:** The top 10 authors for CKD and depression publications.

Rank	Author	Documents	Citations	H Index	Average citation/Publication
1	Kimmel, Paul L	23	1926	84	83.74
2	Weisbord, Steven D	19	1,045	37	55.00
3	Novak, Marta	19	649	33	34.16
4	Dekker, Friedo W	19	223	89	11.74
5	Mucsi, Istvan	17	488	44	28.71
6	Hedayati, S. Susan	15	906	31	60.40
7	Bossola, Maurizio	15	356	41	23.73
8	Unruh, Mark	14	490	36	35.00
9	Cukor, Daniel	12	946	21	78.83
10	Craig, Jonathan c	12	685	98	57.08

**TABLE 2:** The top 10 most cited authors for CKD and depression publications.

Rank	Author	Documents	Citations	H Index	Average citation/Publication
1	Kimmel, Paul L	23	1926	84	83.74
2	Peterson, RA	7	1,221	42	174.43
3	Kimmel, PL	9	1,179	84	131.00
4	Peterson, Rolf A	9	1,049	42	116.56
5	Weisbord, Steven D	19	1,045	37	55.00
6	Cukor, Daniel	12	946	21	78.83
7	Hedayati, S. Susan	15	906	31	60.40
8	Higginson, Irene J	2	780	86	390.00
9	Hays, RD	2	686	115	343.00
10	Craig, Jonathan C	12	685	98	57.08

**FIGURE 3:**
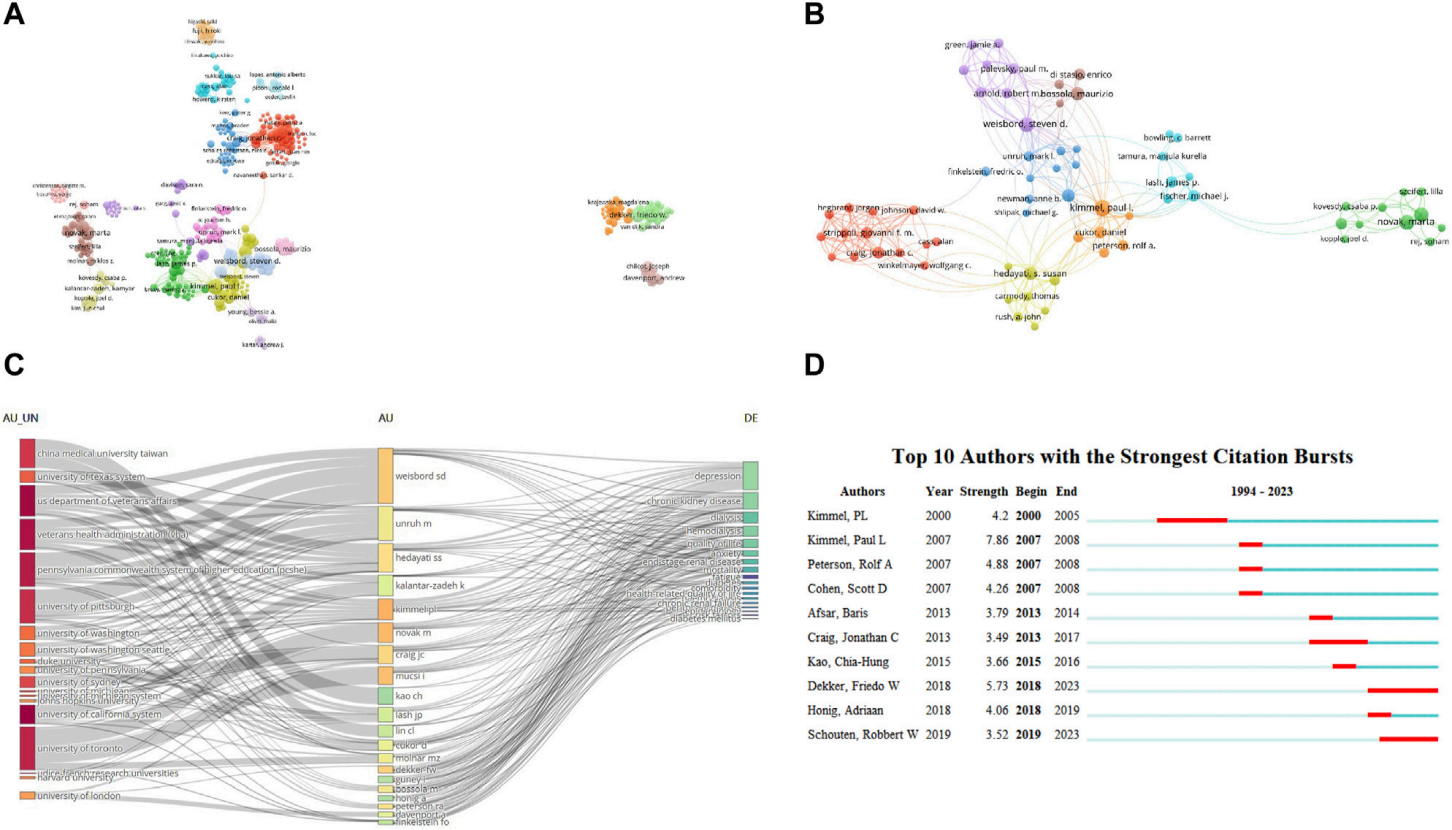
Authors involved in the study of CKD and depression. **(A)** The co-authorship network of cited authors. **(B)** The co-authorship network of productive authors. **(C)** Three-Fields Plot between authors’ institutions (left field), the most productive authors (middle field) and the authors’ keywords (right field). **(D)** Timeline distribution of cluster analysis of top 10 authors.

In the present study, VOSviewer conducted a thorough co-authorship analysis, visualized in Figures 3A, B, illustrating a collaborative network among different authors. Each dot within the network represents an author, with connecting lines denoting collaborative relationships. The color of the connecting lines signifies the nature of collaboration, while the thickness of these lines indicates the intensity of collaboration. Figure 3A displays the co-authorship network of cited authors, revealing intricate connections among 2,580 authors divided into 8 clusters represented by different colors (Figure 3B). The red cluster, centered around authors Strippoli Giovanni F.M, Hegbrant Jorgen, Johnson David W, and Craig Jonathan C, is particularly highlighted. As depicted in Figure 3A, Paul L. Kimmel, the most prolific author in CKD and depression publications, closely collaborated with Daniel Cukor, Rolf A. Peterson, Steven D. Weisbord, and Marta Novak. Similarly, the second and third most productive authors, Weisbord, Steven D, and Novak, Marta, respectively, exhibit close collaborations with their peers. Interestingly, authors with high publication numbers also garner significant citations, suggesting a correlation between productivity and impact. The data suggest that researchers’ cooperation tends to be relatively loose, and the collaborative team operates within a confined scope.

Exploring this section, Figure 3C visually encapsulates the institutions of authors, the connections between authors, and the relationships among keywords. Notably, the keyword “depression” stands out as a central term prevalent across all the analyzed literature from various authors and their respective institutions. Furthermore, an in-depth author burst analysis was conducted, leading to the identification of the top 10 most cited authors, as illustrated in Figure 3D. The interplay of blue and red lines within the timeline reveals the chronological evolution, with the red segment signifying the period of author bursts. Notably, the early years witnessed Kimmel, PL, Peterson, Rolf A, and Cohen, Scott D as the prominent figures in CKD research. However, in the last 5 years, a notable shift occurred, with Dekker, Friedo W, Honig, Adriaan, and Schouten, Robbert W emerging as the frontrunners, capturing the prevailing research trends and attaining the status of the most cited authors.

Table 3 delineates the foremost countries/regions based on research productivity, with the United States emerging as the most prolific nation, contributing 765 publications garnering 29,845 citations. Subsequently, China (216 publications, 3,211 citations), England (168 publications, 5,631 citations), and Canada (145 publications, 5,674 citations) followed in research output strength. Similarly, Table 4 highlights the preeminent countries/regions in terms of citation impact. The United States, with 765 publications, maintained a substantial lead with 29,845 citations. Canada (145 publications, 5,764 citations), England (168 publications, 5,631 citations), and Australia (135 publications, 3,394 citations) secured commendable positions in citation impact. Noteworthy is the observation that while China ranked second in the number of published articles (216 publications), its citation count remained relatively lower at 3,211, suggesting a disparity between research output and impact.

**TABLE 3:** The top 10 productive countries/regions.

Rank	Country/region	Documents	% of (2,409)	Citations	Average citation/Publication
1	United States of America	765	31.76	29,845	39.01
2	Peoples r China	216	8.97	3,211	14.87
3	England	168	6.97	5,631	33.52
4	Canada	145	6.02	5,674	39.13
5	Australia	135	5.60	3,394	25.14
6	Taiwan	130	5.40	2,277	17.52
7	Turkey	127	5.27	1961	15.44
8	Italy	127	5.27	3,334	26.25
9	Germany	97	4.03	2,433	25.08
10	Netherlands	94	3.90	2,948	31.36

USA, the United States of America; Peoples r China, the People’s Republic of China.

**TABLE 4:** The top 10 most cited countries/regions.

Rank	Country/region	Documents	% Of (2,409)	Citations	Average citation/Publication
1	United States of America	765	31.76	29,845	39.01
2	Canada	145	6.02	5,674	39.13
3	England	168	6.97	5,631	33.52
4	Australia	135	5.60	3,394	25.14
5	Italy	127	5.27	3,334	26.25
6	Peoples r China	216	8.97	3,211	14.87
7	Netherlands	94	3.90	2,948	31.36
8	Brazil	90	3.74	2,502	27.80
9	Germany	97	4.03	2,433	25.08
10	France	52	2.16	2,301	44.25

USA, the United States of America; Peoples r China, the People’s Republic of China.

**FIGURE 4:**
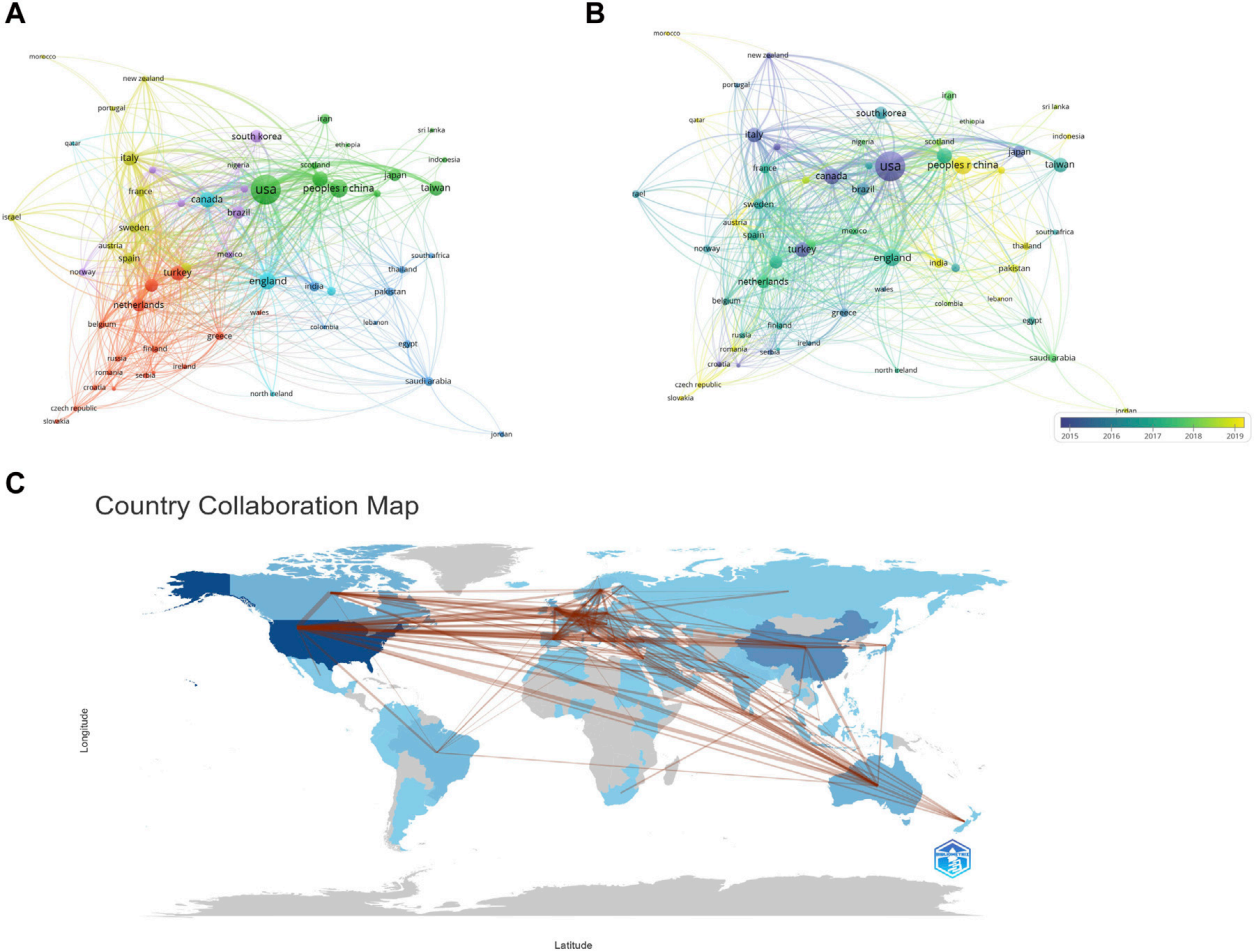
Countries contribution and collaboration. **(A)** Countries collaboration map by using Vosviewer. **(B)** Countries collaboration map between 2015 and 2019 by using Vosviewer. **(C)** Countries contribution of CKD and depression based on total publications and collaboration between contributing countries by using biblioshiny.

VOSviewer was employed to conduct a comprehensive co-authorship analysis of countries/regions, shedding light on the intricate landscape of international collaborations in the field. The resulting collaboration map, depicted in Figure 4, illustrates the relationships among countries engaged in CKD research. The interconnected lines between two countries signify the extent of their cooperation, with the thickness and quantity of the lines reflecting the depth and frequency of collaboration. Notably, the collaborative map reveals robust engagement among the United States, China, and Western European countries, underscoring their prominent roles in international research collaboration within the CKD domain. Furthermore, the United States emerges as a central hub, boasting the highest number of collaborative endeavors with other nations, accentuating its leadership in fostering international research partnerships.

### Distribution and co-authorship of institutions

Table 5 presents the top 10 institutions with the highest productivity in terms of CKD publications. The University of Pittsburgh, with 52 publications and 3,732 citations, leads in output, followed by the University of Toronto (47 publications, 2,154 citations), Duke University (47 publications, 1,105 citations), University of Michigan (46 publications, 1,898 citations), and University of California-San Francisco (46 publications, 2,246 citations). Table 6 outlines the top 10 most cited institutions in CKD research, with citation counts ranging from 1,279 to 3,732. Notably, the University of Pittsburgh emerges as the most cited institution (3,732 citations), securing the top position in the list. Following closely are George Washington University (3,716 citations), University of California-San Francisco (2,246 citations), and University of Toronto (1,179 citations). Significantly, the University of Pittsburgh stands out as both the most productive and most cited institution, underscoring its prominent role in CKD research.

**TABLE 5:** The top 10 productive institutions.

Rank	Orgnizations	Documents	Citations	Average citation/Publication
1	Univ Pittsburgh	52	3,732	71.77
2	Univ Toronto	47	2,154	45.83
3	Duke Univ	47	1,105	23.51
4	Univ Michigan	46	1898	41.26
5	Univ Calif San Francisco	46	2,246	48.83
6	Univ Washington	39	1,349	34.59
7	Univ Sydney	36	1,166	32.39
8	George Washington Univ	35	3,716	106.17
9	China Med Univ	34	429	12.62
10	Johns Hopkins Univ	30	1,313	43.77

Univ Pittsburgh, University of Pittsburgh; Univ Toronto, University of Toronto; Duke Univ, Duke University; Univ Michigan, University of Michigan; Univ Calif San Francisco, University of California-San Francisco; Univ Washington, University of Washington; Univ Sydney, University of Sydney; George Washington Univ, George Washington University; China Med Univ, China Medical University; Johns Hopkins Univ, Johns Hopkins University.

**TABLE 6:** The top 10 cited institutions.

Rank	Orgnizations	Documents	Citations	Average citation/Publication
1	Univ Pittsburgh	52	3,732	71.77
2	George Washington Univ	35	3,716	106.17
3	Univ Calif San Francisco	46	2,246	48.83
4	Univ Toronto	47	2,154	45.83
5	Univ Michigan	46	1898	41.26
6	Washington Univ	7	1,374	196.29
7	Univ Washington	39	1,349	34.59
8	Univ Alberta	25	1,348	53.92
9	Johns Hopkins Univ	30	1,313	43.77
10	Va Pittsburg Healthcare Syst	20	1,279	63.95

Univ Pittsburgh, University of Pittsburgh; George Washington Univ, George Washington University; Univ Calif San Francisco, University of California-San Francisco; Univ Toronto, University of Toronto; Univ Michigan, University Michigan; Washington Univ, Washington University; Univ Washington, University of Washington; Univ Alberta, University of Alberta; Johns Hopkins Univ, Johns Hopkins University; Va Pittsburg Healthcare Syst, Pittsburgh Health Care System.

**FIGURE 5:**
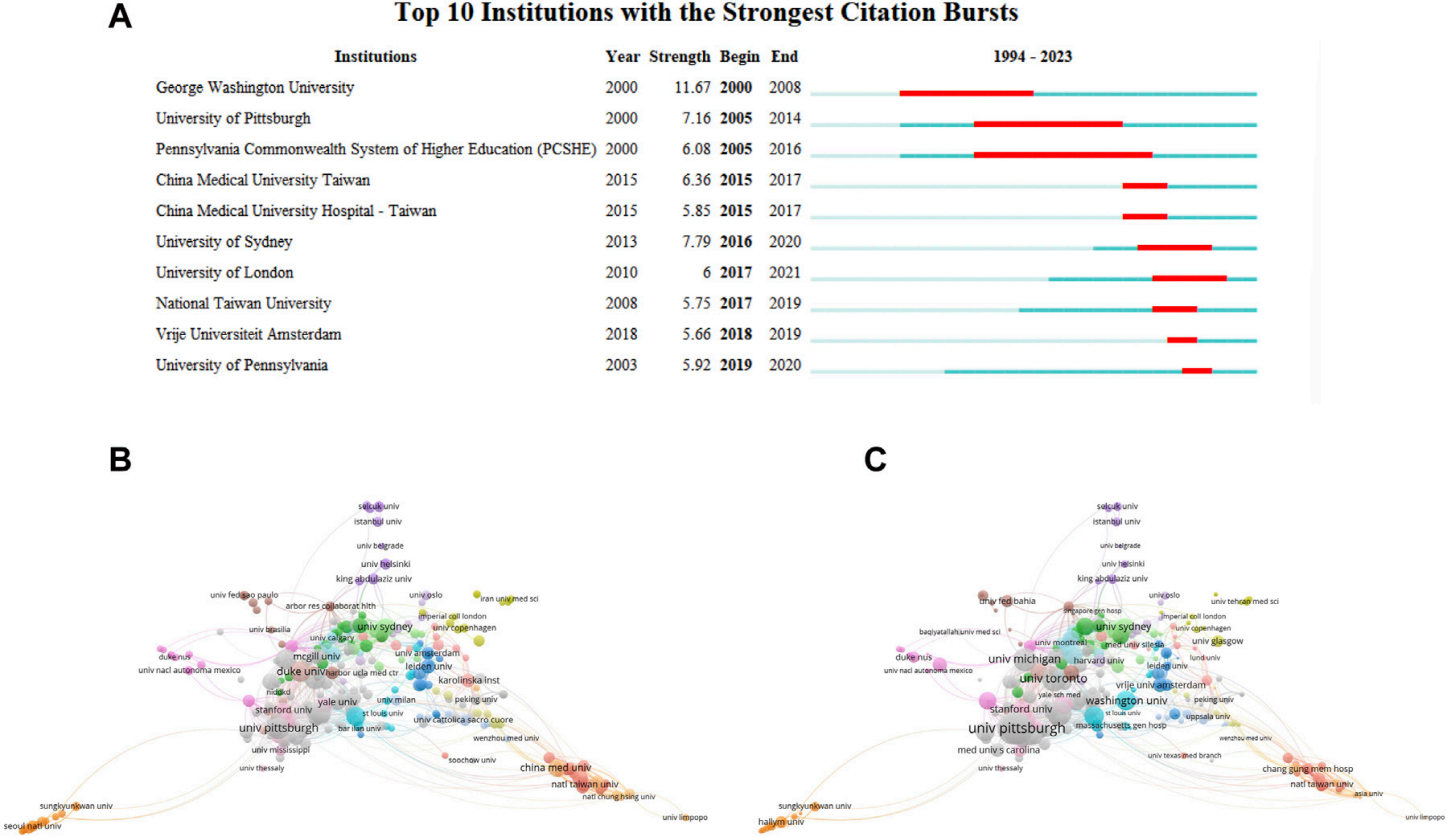
Institutions contribution and collaboration. **(A)** Timeline distribution of cluster analysis of institutions. **(B)** Productive institutions collaboration map by using Vosviewer. **(C)** The top cited institutions collaboration map by using Vosviewer.

The institutions exhibiting the most pronounced citation bursts are depicted in Figure 5A. Notably, George Washington University held the leading position in citations during the period spanning 2000 to 2008. Contrarily, the University of Pittsburgh secured the third rank, trailing behind the University of Sydney. Figures 5B, C present collaboration maps illustrating the relationships among institutions engaged in CKD research. These visualizations provide a comprehensive overview of collaborative efforts within the CKD domain.

**FIGURE 6:**
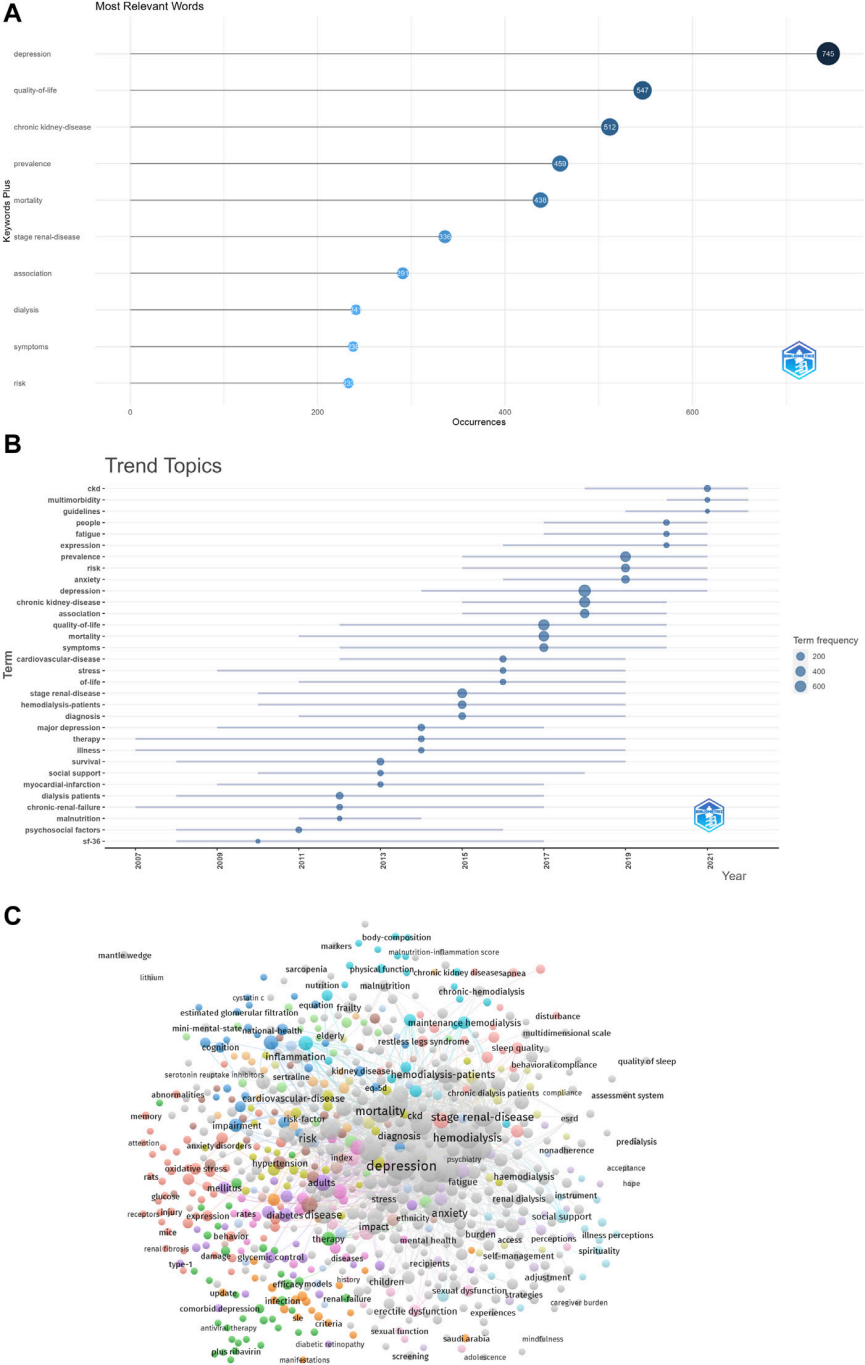
The contribution of keywords. **(A)** The top 10 most relevant keywords. **(B)** Trends in terminology over the years. **(C)** The co-occurrence analysis map of the keywords about CKD.

Keywords serve as pivotal indicators of the primary themes in publications, making high-frequency keywords ideal candidates for co-occurrence analysis. In the current study, VOSviewer was employed to extract and cluster the top 10 most relevant keywords, as outlined in Table 7. The trends in terminology evolution over the years are visually presented in Figures 6A, B, revealing a growing popularity in CKD research. Figure 6C provides a co-occurrence analysis map of CKD-related keywords. The node labels represent specific keywords, and the node size correlates with the frequency of each keyword. Links connecting nodes signify co-occurrence relationships between respective keywords. Notably, the central position in the visualization network map is occupied by keywords such as depression, hemodialysis, mortality, stage renal-disease, and CKD, indicating their significance in the co-occurrence analysis. In summary, CKD research has emerged as a prominent and popular topic in recent years, with depression standing out as the most relevant keyword in the field.

**TABLE 7:** The top 10 most relevant keywords.

Rank	Keyword	Count
1	Depression	1,225
2	Quality-of-life	549
3	Mortality	513
4	Chronic kidney-disease	512
5	Prevalence	474
6	Hemodialysis	440
7	Dialysis	370
8	Chronic kidney disease	347
9	Stage renal-disease	336
10	Anxiety	317

### The analysis of journals

**FIGURE 7:**
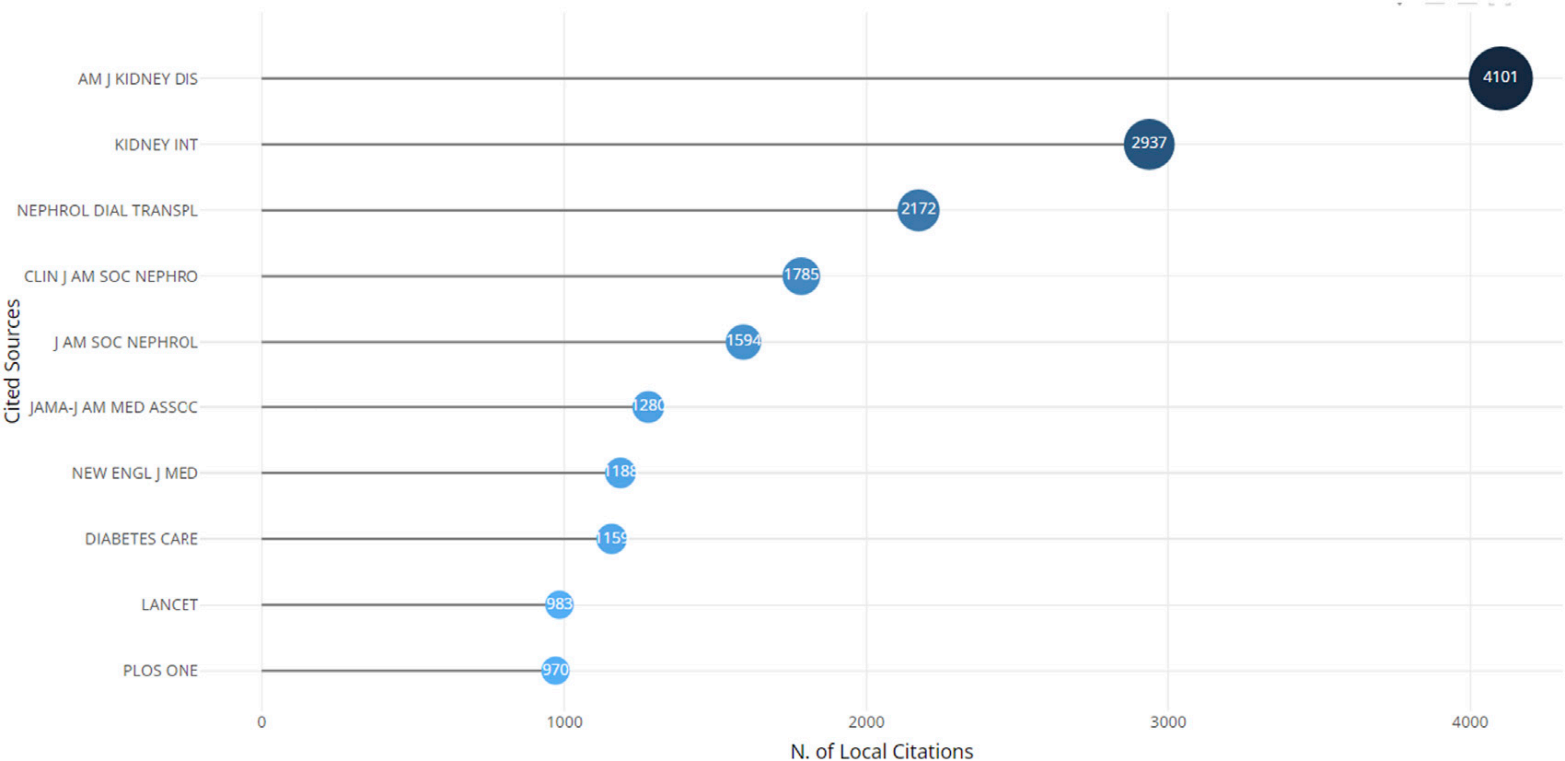
The top 10 most cited journal.

Figure 7; Tables 8, 9 delineate the top 10 most productive and most cited journals, respectively, in the field of CKD. BMC Nephrology, boasting an impact factor of 2.3 and a Journal Citation Reports (JCR) Q3 designation, emerged as the most prolific publisher, contributing 2.03% of all CKD publications. Following closely were the American Journal of Kidney Diseases (1.99%), PLOS ONE (1.87%), and the Clinical Journal of the American Society of Nephrology (1.83%). In the realm of the most frequently cited journals, the American Journal of Kidney Diseases claimed the top spot with 3,171 citations, holding an impressive impact factor of 13.2 and a JCR Q1 designation. Notably, 70% of the top 10 most cited journals were categorized as Q1, while the remaining 30% fell within Q2 or Q3.

**TABLE 8:** The top 10 productive publication journal.

Rank	Publication titles	Documents	%of (2,562)	Citations	Average citation/Publication
1	Bmc nephrology	52	2.03	959	18.44
2	American journal of kidney diseases	51	1.99	3,171	62.18
3	Plos one	48	1.87	1,039	21.65
4	Clinical journal of the American society of nephrology	47	1.83	2,915	62.02
5	Nephrology dialysis transplantation	44	1.72	2,251	51.16
6	Renal failure	40	1.56	643	16.08
7	International urology and nephrology	35	1.37	463	13.23
8	Bmj open	31	1.21	389	12.55
9	International journal of environmental research and public health	28	1.09	119	4.25
10	Journal of psychosomatic research	27	1.05	884	32.74

**TABLE 9:** The top 10 most cited publication journal.

Rank	Journal	IF/JCR (2023)	Citations
1	American journal of kidney diseases	13.2/Q1	3,171
2	Clinical journal of the American society of nephrology	9.8/Q1	2,915
3	Kidney international	19.6/Q1	2,838
4	Nephrology dialysis transplantation	6.1/Q1	2,251
5	Journal of the American society of nephrology	13.6/Q1	2,143
6	Psychosomatic medicine	3.3/Q3	1,498
7	Advances in chronic kidney disease	2.9/Q1	1,054
8	Plos one	3.7/Q2	1,039
9	Bmc nephrology	2.3/Q3	959
10	Gastroenterology	29.4/Q1	882

IF/JCR, Impact Factor/Journal Citation Reports.

Table 10 displays the predominant paper categories, with the primary focus falling within urology nephrology, general internal medicine, psychiatry, occupational and environmental health, healthcare science services, nursing, transplantation, endocrinology metabolism, clinical neurology, and pharmacology pharmacy.

**TABLE 10:** The top 10 most popular types of papers.

Rank	The main types of papers	Count
1	Urology nephrology	673
2	Medicine general internal	337
3	Psychiatry	209
4	Public environmental occupational health	166
5	Healthcare science services	134
6	Nursing	108
7	Transplantation	106
8	Endocrinology metabolism	104
9	Clinical neurology	89
10	Pharmacology pharmacy	88

**FIGURE 8:**
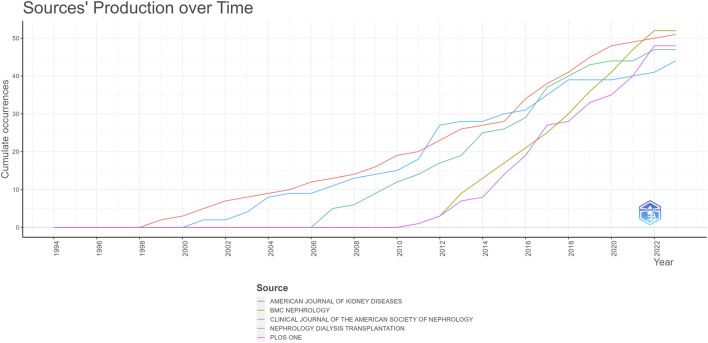
Top 5 journals publications trends overtime by using biblioshiny.

Examining the upward trajectory depicted in Figure 8 for the leading five journals, the American Journal of Kidney Diseases has consistently maintained its prominence. However, notable is the rapid surge experienced by BMC Nephrology in recent years. As of 2022, the cumulative volume of publications in BMC Nephrology has surpassed that of the American Journal of Kidney Diseases, and this trend is persistently on the upswing. This suggests a noteworthy ascension in research activity within the journal, marking it as a burgeoning hub for scholarly contributions in the field.

## Discussion

In this study, we conducted a bibliometric analysis of research on depression and CKD using the Web of Science database, CiteSpace and VOSviewer software to identify major themes and emerging trends. Our analysis specifically focused on the relationship between depression and CKD and revealed a total of 12,761 authors who published 2,409 articles between 2006 and 2022. In recent years, there has been a growing interest in the field of depression and CKD, as evidenced by the increasing number of research articles on this topic. The number of articles steadily rose from 2006 to 2016, and saw a significant increase since 2016. Although the number of published articles decreased slightly in 2021, it regained momentum in 2022 reaching a peak.

Furthermore, lots of studies mainly focused on the association between CKD and depression, clinical outcomes and quality of life of the patients with CKD (Alshelleh et al., 2023; Huang et al., 2023; Zhu et al., 2023), which suggesting that there have been growing concerns on CKD with depression in the last decade. However, researches related to the treatment of CKD in combination with depression remains insufficient. It is anticipated that this area of research will continue to gain attention moving forward.

The United States stands at the forefront of CKD research, bolstered by substantial resources, robust collaborations, and strategic partnerships. Between 2006 and 2022, it led the world in both the quantity of scholarly articles published on CKD and depression and the number of citations received. This prominence is closely linked to its economic prowess and substantial healthcare spending. Reports indicate that in 2016, the United States topped global health expenditure charts, investing an average of $9,892 per citizen, a stark contrast to the average healthcare spending in many low-income countries, which barely exceeds $100 per person annually (Dieleman et al., 2018; Onarheim et al., 2018). This disparity in healthcare investment is a significant factor contributing to the United States’ dominance in CKD research and publication output.

An analysis of collaborative networks indicates partnerships between various countries. The majority of collaborative efforts in CKD and depression research are concentrated in the United States, aligning with the country’s prominent role in this field of study. However, our analysis highlights a significant gap in collaborative efforts with several regions. Notably, countries like Morocco, Jordan, Slovakia, and Sri Lanka show limited involvement in these global research partnerships. This disparity may be attributed to various factors, including differences in research funding, infrastructure, and priorities in healthcare. The underrepresentation of these countries in global research collaborations underscores the need for a more inclusive approach that encourages and supports cross-border research efforts. Fostering a more equitable and diverse research network is not only essential for advancing scientific knowledge but also for ensuring that the benefits of research are shared globally. This calls for concerted efforts from leading research nations, international health organizations, and funding agencies to bridge these gaps and build stronger, more inclusive research networks.

We analyzed authors by examining not only their article and citation count but also their H-index. Both European and American researchers have a rich history of research in this field and have achieved significant progress at an early stage. For instance, Kimmel, Paul L, a professor of Clinical Medicine at George Washington University, has authored books on nephrology and played a significant role in studying the etiology, diagnosis, and treatment of CKD. Additionally, in recent years, Friedo W. Dekker, Robbert W. Schouten, and others have also explored the latest research topics and have become the most cited authors. Dekker and Schouten have conducted research on survival rates, quality of life, and disease progression among CKD patients on peritoneal dialysis (Ocak et al., 2010; Haverkamp et al., 2019; Schouten et al., 2019; Eveleens et al., 2022).

Bibliometrics plays a vital role in sorting out significant trends and VOSviewer is aim to examine hot topic patterns to identify influential articles and assess diverse research directions objectively. Recently, CKD has gained attention as a popular topic, emerging around 2018 and resulting in 512 publications in 1 year. Among those papers, hot topics included quality of life, mortality, prevalence and therapy on CKD with depression (Hedayati et al., 2012; Haarhaus et al., 2023). However, there is a limited amount of literature on multimorbidity and guidelines in this field (Burrows et al., 2022; Chinese Experts Group of the Guideline for the Management of ‘CKD-PeriDialysis’Chinese Non-government Medical Institutions Association, 2022). Nonetheless, these topics have gained less popularity in the past few years, indicating potential for emerging research areas in the future.

Among the leading journals in this field, BMC Nephrology and the American Journal of Kidney Diseases have published the highest number of articles, while others such as Neurology, Nephrology, Medicine General Internal, and Psychiatry have also contributed to this area of study. Of the top 10 journals with the highest number of citations for CKD and depression related studies, 6 journals with an impact factor exceeding 10 were identified. The proportion of articles published in these journals is less than 5% of the total number of publications. Hence, it is a challenge to publish high-quality papers about CKD and depression-related research in distinguished journals. To date, only a few articles have been published in journals with high impact factors, implying that such journals have carried out more innovative research in this field. Therefore researchers who are interested in this area can concentrate on the most recent publications in these journals.

Chronic Kidney Disease (CKD) and depression are closely linked, with numerous studies highlighting the bidirectional relationship between the two conditions. Patients with CKD are at a higher risk of developing depression due to the chronic nature of the disease, frequent hospital visits, and the associated physical and psychological burden. Conversely, depression can negatively impact the prognosis of CKD by influencing patients’ adherence to treatment and lifestyle modifications.

Recent research suggests that the inflammatory processes involved in CKD may also contribute to the development of depression. Elevated levels of pro-inflammatory cytokines in CKD patients have been associated with depressive symptoms. Additionally, the burden of CKD management, including dietary restrictions and dialysis, can further exacerbate mental health issues. However, the causal link between depression and CKD is unclear. One study found that high depressive symptoms occurred in participants with normal renal function, which was significantly linked to a high risk of rapid decline in renal function at baseline (Zhang et al., 2021), while another study found no significant association between depressive symptoms and rapid decline in eGFR in participants without CKD at baseline (Kop et al., 2011).

Addressing depression in CKD patients is crucial for improving their overall quality of life and clinical outcomes. Integrated care approaches that include psychological support and interventions aimed at managing both CKD and depression are essential. Future research should continue to explore the mechanisms underlying the CKD-depression link and develop targeted therapies to address this comorbidity.

## Limitations

This study has some limitations. First, the analysis of this study is based on quantitative data, which may not fully capture the qualitative aspects of research impact and significance. It can be biased by factors such as self-citations and the varying citation practices across different disciplines. Second, this article only includes English articles, which reduces the number of articles that were retrieved. Finally, it may overlook emerging research about CKD and depression that have not yet accumulated significant citations, potentially leading to an incomplete or skewed understanding of research trends and contributions.

## Conclusion

To date, there has been no comprehensive assessment of the evidence on depression and CKD, and our searches did not identify any ongoing bibliometric studies on this topic. This study clarifies the correlation between depression and CKD, emphasizing the impact of depression on the risk of psychiatric factors and comorbidities in patients with CKD. Patients diagnosed with CKD are frequently linked to a range of psychosomatic abnormalities. Depression can exacerbate CKD and lead to additional health problems. Studies on this topic have increased since 2006, indicating growing interest in this area. Based on bibliometric data, the United States, China, and the United Kingdom are leading contributors to this field. Timely diagnosis and treatment of depression is crucial for CKD patients to prevent disease progression. These developing patterns emphasize the necessity for additional research to enhance our knowledge and control of depression in the context of CKD.
